# Distribution of multi-drug resistant tuberculosis in Ekiti and Ondo states, Nigeria

**DOI:** 10.1016/j.nmni.2023.101192

**Published:** 2023-10-31

**Authors:** Olugbenga Enoch Olabiyi, Pius Abimbola Okiki, Mumuni Idowu Adarabioyo, Oludele Emmanuel Adebiyi, Olusola Emannuel Adegoke, Olubunmi Ebenezer Esan, Olayinka O. Idris, Toluwani Bosede Agunbiade

**Affiliations:** a Department of Biological Sciences, Afe Babalola University, Ado-Ekiti, Nigeria; b Department of Medical Microbiology and Parasitology, Ekiti State Teaching Hospital, Ado-Ekiti, Nigeria; c Institute of One-Health, Afe Babalola University, Ado-Ekiti, Nigeria; d Department of Mathematical & Physical Sciences (Statistics Unit), Afe Babalola University, Ado-Ekiti, Nigeria; e South-West Zonal Tuberculosis Reference Laboratory, Department of Medical Microbiology and Parasitology, University College Hospital, Ibadan, Nigeria; f Department of Medical Microbiology and Parasitology, Ondo State Specialist Hospital, Akure, Nigeria; g Department of Integrated General Medical Sciences, Afe Babalola University, Ado-Ekiti, Nigeria

**Keywords:** Antimycobacterial agents, Infectious disease *Mycobacterium tuberculosis*, Rifampicin-resistant, Tuberculosis

## Abstract

**Background:**

Tuberculosis (TB), caused by *Mycobacterium tuberculosis* (MTB), is one of the top infectious killer diseases in the world. The emergence of drug-resistant MTB strains has thrown challenges in controlling TB worldwide. This study investigated the prevalence of drug-resistant tuberculosis in the states of Nigeria and the risk factors that can increase the incidence of tuberculosis.

**Methods:**

The study is a cross-sectional epidemiological research carried out in the six senatorial districts of Ekiti and Ondo states, Nigeria, between February 2019 and January 2020. A structured questionnaire was administered to 1203 respondents for socio-demographic information, and sputum samples were collected from them for TB investigation. GeneXpert technique was used to diagnose TB from the sputum samples, followed by bacterial isolation using Löweinstein-Jensen medium and antibiotic susceptibility testing.

**Results:**

Prevalence of TB in the two states combined was 15 ​%; with 13.8 ​% for Ekiti state and 16.1 ​% for Ondo State. The distribution of TB in the senatorial districts was such that: Ondo South ​> ​Ekiti Central ​> ​Ekiti South ​> ​Ondo North ​> ​Ekiti North ​> ​Ondo Central. The risk factors identified for TB prevalence in two states were gender, male ​> ​female (OR ​= ​0.548, p ​= ​0.004); overcrowding (OR ​= ​0.733, p ​= ​0.026); room size (OR ​= ​0.580, p ​= ​0.002); smoking (OR ​= ​0.682, p ​= ​0.019) and dry and dusty season (OR ​= ​0.468, p ​= ​0.005). The prevalence of MDR-TB in Ekiti and Ondo States were 1.2 ​% and 1.3 ​% respectively. The identified risk factors for MDR were education (OR ​= ​0.739, p ​= ​0.017), age (OR ​= ​0.846, p ​= ​0.048), religion (OR ​= ​1.95, p ​= ​0.0003), family income (OR ​= ​1.76, p ​= ​0.008), previous TB treatment (OR ​= ​3.64, p ​= ​0.004), smoking (OR ​= ​1.33, p ​= ​0.035) and HIV status (OR ​= ​1.85, p ​= ​0.006). Rifampicin monoresistant was reported in 6.7 ​% of the rifampicin-resistant strains, while 93.3 ​% were rifampicin polyresistant strains. Two (13.3 ​%) of the MDR-TB strains were resistant to all the 3 first-line antimycobacterial agents. All the Rifampicin-resistant TB strains were susceptible to the aminoglycosides (Amikacin, Capreomycin and Kanamycin), also with high susceptibility to the fluoroquinilones: Moxifloxacin (100 ​%) and Levofloxacin (86.7 ​%). Sixteen (94.1 ​%) of the 17 Rifampicin-susceptible strains were susceptible to all the eight antibiotics tested, while one (5.9 ​%) was susceptible to Rifampicin and Isoniazid but resistant to the rest antibiotics. Conclusion: The study showed that there is high prevalence of TB and MDR-TB in Ekiti and Ondo States Nigeria, hence, to meet the SDG Target 3.3 of ending TB epidemic by 2030, culturing and antibiotic susceptibility testing should be carried out on every TB-positive sputum and the patients treated accordingly.

## 1 Background

Tuberculosis (TB) is one of the top ten causes of death worldwide, as well as the second-leading pathogenic agent-related cause of death, behind HIV/AIDS [1]. *Mycobacterium tuberculosis* (MTB) is the causative agent of TB, and the disease spreads when an infected individual coughs, thereby releasing an aerosol containing the active bacteria into the air which is inhaled by susceptible individuals [1,2]. Infection occurs when the inhaled bacteria become active due to some immunosuppressive conditions such as old age, diabetes, and HIV infection. Majorly, the lung is affected (pulmonary TB), but other body organs may as well be affected (extrapulmonary TB) [1].

Multidrug-resistance TB (MDR-TB) is a type of TB that is resistant to the first-generation drugs used for treating TB (rifampicin plus isoniazid or other first-line drugs); TB caused by *M. tuberculosis* strains that fulfill the definition of MDR/RR-TB and which are also resistant to any fluoroquinolone and at least one additional Group A drug (Group A drugs are the most potent group of drugs in the ranking of second-line medicines for the treatment of drug-resistant forms of TB using longer treatment regimens and comprise levofloxacin, moxifloxacin, bedaquiline, and linezolid) is called extensively drug-resistant TB (XDR-TB) [3]. *M. tuberculosis*, like other bacteria, develops resistance to drugs as a result of genetic changes such as mutations [4]. Inappropriate treatment of TB frequently results in the development and spread of multidrug-resistant tuberculosis [1]. Such inappropriate treatment of TB could be a result of incorrect medications, substandard medication (standard treatment includes at least two drugs), inconsistent medication, or failure to complete the treatment period (which is often required for several months) [5,6]. Some social determinants responsible for the development of multi-drug resistant TB include inadequate resources for treatment, high poverty level, poor living standard, and various causes of social vulnerability and non-availability of quality health services [7]. In addition, social-related behaviour factors in individuals and communities like smoking, alcoholism, and overcrowding are considered risk factors [1]. In most cases TB is treatable and curable, however poor clinical outcomes are associated with improper treatment [8].

**Fig. 1:**
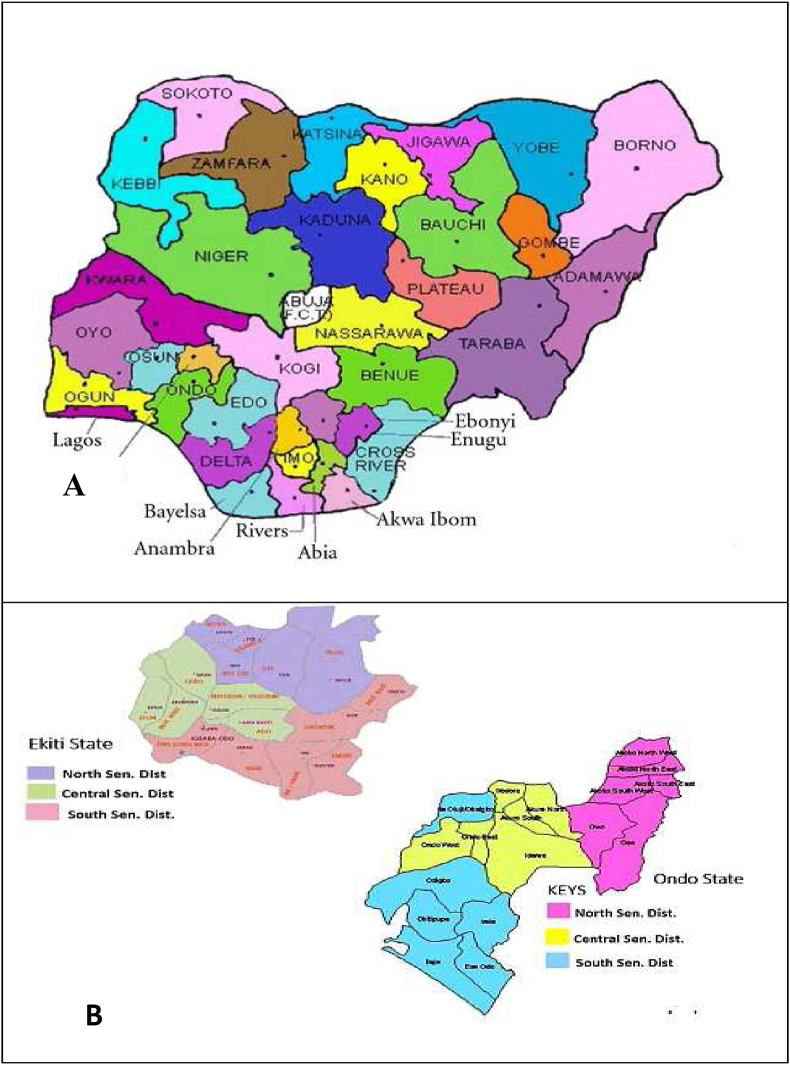
Map of Nigeria showing geographical relationship between Ekiti and Ondo States (A) and the administrative maps showing senatorial districts of both Ekiti and Ondo States (B). Source: Google maps.

Timely diagnosis of TB and adequate treatment is one of the ways to curb the spreading of this infectious disease and its development of resistant strains. In 2020, 71 ​% of people worldwide with pulmonary TB were bacteriologically confirmed rifampicin-resistant TB; an increase in 2018 and 2019 global figures [1]. Treatment of MDR-TB requires second-line anti-tuberculosis medication, such as fluoroquinolones and aminoglycosides, although some of these drugs have been reported to be more toxic and expensive than first-line drugs [9]. Aside from this, patients infected with MDR strains can develop drug-related complications especially when they are co-infected with HIV or suffer from other immune-suppressive diseases [10]. Such patients often undergo long periods of treatment, extensive chemotherapy, psychological problems, economic wastage, poor treatment outcomes, further resistance leading to XDR-TB [[11], [12], [13]]; as well as higher case fatality rates due to drug toxicity [11,12].

This study investigated the prevalence of drug-resistant tuberculosis in the states of Nigeria and risk factors which can increase the incidence of tuberculosis.

## 2 Methods

### 2.1 Study design

The study was conducted at directly observed therapy of TB (TB-DOT) centres in Ekiti and Ondo States, Nigeria, between February 2019 and January 2020. Three centres were selected in each state, one centre per senatorial district. All the selected TB-DOTS centres were well-equipped with facilities for the diagnosis and management of tuberculosis.

Ekiti and Ondo states used to be one and the same Ondo state, up to 1999 when Ekiti state was carved out. The two states are linked together in socio-cultural and infrastructural like road networks, airports, and electricity; also share common river bodies. Within the two states, there is a lot of migration through education, job opportunities, commerce and industry, agriculture, and many more. Each of the states is composed of three senatorial districts (Fig. 1).

### 2.2 Data collection

A total of 1203 patients who satisfied the inclusion criteria were recruited for the study. The socio-demographics and clinical history were obtained using a structured questionnaire that was administered in the DOTS centre to the patients. The clinical information obtained from the participants includes reported symptoms and duration, previous treatment for TB, contact with TB patients, smoking habits, alcohol intake, non-communicable disease, and HIV/AIDS Status.

### 2.3 Sputum collection

On-spot sputum samples were collected from each of the participants, for TB investigation.

### 2.4 Detection of MTB and MTB/RIF

The sputum specimens obtained were analyzed using the GeneXpert MTB/RIF® (Cephied, Sunnyvale, CA) to detect TB and MDR-TB. GeneXpert MTB/RIF provides rapid and sensitive detection of TB and rifampicin resistance using the principle of real-time polymerase chain reaction [1,14]. The protocol of the Genexpert MTB/RIF machine was strictly followed.

### 2.5 Isolation of MTB from sputum

Culturing of sputum specimens for isolation of MTB was carried out on GeneXper-confirmed 15 rifampicin-resistant and randomly selected 17 rifampicin-susceptible sputum samples were cultured on Löweinstein-Jensen (LJ) solid culture medium, incubated at 37 ​°C for 6–8 weeks for isolation of *M. tuberculosis* [15,16].

### 2.6 Susceptibility to anti-mycobacterium agents

A suspension of isolated *M. tuberculosis* was made by taking a loopful of mycobacterial culture from LJ medium and placing it in a sterile tube containing 1 ​mL of sterile distilled water and 3 ​mm diameter of 6 glass beads. The tube was vortexed for 20–30 ​s and the opacity of the bacterial suspension was then adjusted by the addition of distilled water to obtain a concentration of 1 ​mg/mL of tubercule bacilli by matching the McFarland standard No.1. After preparing the standard neat suspension, one or two loop-full of the suspension is added to each LJ medium containing antibiotic of concentration 2 ​μg/mL the medium were incubated at 37 ​°C and were read at the 4th and 6th weeks [17]. The antibiotics tested were Isoniazid (INH), Ethambutol (EMB), Kanamycin (KAN), Amikacin (AMK), Capreomycin (CAP), Levofloxacin (LEV), Moxifloxacin (MOX) and Protionamide/Ethionamide (PRO/ETO).

The isolation of *M. tuberculosis* and the susceptibility of the isolates to anti-mycobacterial agents was carried out at the TB zonal reference laboratory, University Teaching Hospital, Ibadan, Nigeria, which maintains strict external control. The laboratory is the first National Tuberculosis Reference Laboratory in Nigeria and is domiciled within the Centre for Tuberculosis Research (CTBR). This Centre contributes to the WHO End TB Strategy programme through research, service, and training. CTBR conducts a range of multi-disciplinary research and training by dedicated scientists and support staff in collaboration with universities, and public and private sector organizations within the sub-region and internationally.

### 2.7 Data analysis

All data generated from the studied population were analyzed using the IBM SPSS Statistics for Windows, Version 20.0. Armonk, NY: IBM Corp. (IBM SPSS, 2012), to determine the frequency of the demographic variables. Logistic regression was used to determine the association between tuberculosis prevalence and relevant variables using R version 4.2.0 (Vigorous Calisthenics) and values of P ​≤ ​0.05 were considered statistically significant.

## 3 Results

Total respondent in the TB investigation was 1203, made up of 587 (48.8 ​%) and 616 (51.2 ​%) from Ekiti and Ondo States respectively. The combined prevalence of TB in the two states was 15 ​%, with Ekiti state having 13.8 ​% and Ondo state 16.2 ​%. The distribution of TB in the senatorial districts was such that: Ondo South ​> ​Ekiti Central ​> ​Ekiti South ​> ​Ondo North ​> ​Ekiti North ​> ​Ondo Central. The prevalence of MDR-TB, identified as rifampicin resistance, in Ekiti and Ondo States were 1.2 ​% and 1.3 ​% respectively; high occurrences of MDR-TB were recorded in Ekiti North and Ondo Central Senatorial Districts (Table 1).

**Table 1:** Distribution frequency of TB and MDR-TB along the senatorial districts in Ekiti and Ondo States, Nigeria.

State	Senatorial District	Total	TB Negative (%)	TB Positive (%)	MDR-TB/population (%)	MDR-TB per TB Positive (%)
Ekiti	Ekiti North	96	88 (91.6 %)	8 (8.4 %)	3 (3.1)	37.5
	Ekiti Central	378	320 (84.6 %)	58 (15.4 %)	3 (0.8)	5.2
	Ekiti South	113	98 (86.7 %)	15 (13.3 %)	1 (0.9)	6.7
	Sub-Total	587	506 (86.2 %)	81 (13.8 %)	7 (1.2)	8.6
Ondo	Ondo North	163	142 (87.1 %)	21 (12.9 %)	0 (0.0)	0
	Ondo Central	278	264 (94.9 %)	14 (5.1 %)	5 (1.8)	35.7
	Ondo South	175	111 (63.4 %)	64 (36.4 %)	3 (1.7)	4.7
	Sub-Total	616	517 (83.9 %)	99 (16.1 %)	8 (1.3)	8.1
Ekiti & Ondo	Total	1203	1023 (85 %)	180 (15 %)	15 (1.3)	7.9

TB – Tuberculosis MDR-TB – Multidrug Resistant Tuberculosis.

Young people of 20 years and below recorded lower TB prevalence (11 ​%) than people above 20 years of age (15.7 ​%), and the age group at risk was 51–60 years with 25.9 ​% TB prevalence. A significantly higher TB occurrence was reported among males (19.1 ​%) than the females (11.0 ​%) participants; with a male-to-female ratio of 1.7:1. Individuals with no formal education reported higher TB occurrence (43 of 167; 25.7 ​%) than those with formal education (137 of 1036; 13.2 ​%). Individuals with divorced marital status had the highest TB occurrence of 22.5 ​% compared to 10.3–17.2 ​% for other categories of marital status. Religion was not found to have a significant association with TB occurrence; however, participants practicing the Islamic faith reported higher TB prevalence (22.5 ​%) than those practicing other religions (12.5–13.2 ​%). Unemployed individuals and low-income earners reported higher TB prevalence than employed and high-income earners, respectively (Table 2).

**Table 2:** Association of tuberculosis with demographic indices in Ekiti and Ondo States, Nigeria.

Characteristics		Total (%)	TB Negative (%)	TB Positive (%)	Odd Ratio	Lower Odd Ratio	Upper Odd Ratio	*P*-Value
Age	≤10	70 (5.8)	63 (90.0)	7 (10.0)	1.0082	0.9328	1.0898	0.9160
	11–20	121 (10.1)	107 (88.4)	14 (11.6)				
	21–30	252 (21.0)	223 (88.5)	29 (11.5)				
	31–40	270 (22.4)	220 (81.5)	50 (18.5)				
	41–50	188 (15.6)	162 (86.2)	26 (13.8)				
	51–60	158 (13.1)	117 (74.1)	41 (25.1)				
	>60	144 (12.0)	131 (91.0)	13 (9.0)				
Gender	Male	593 (49.3)	480 (80.9)	113 (19.1)	0.5484	0.4458	0.6747	0.0037∗∗
	Female	610 (51.7)	543 (89.0)	67 (11.0)				
Education	No formal	167 (13.9)	124 (74.3)	43 (25.7)	0.9017	0.8016	1.0142	0.3790
	Primary	226 (18.8)	200 (88.5)	26 (11.5)				
	Secondary	472 (39.2)	408 (86.4)	64 (13.6)				
	Tertiary	338 (28.1)	291 (86.1)	47 (13.9)				
Marital Status	Single	399 (33.2)	358 (89.7)	41 (10.3)	1.1094	0.8805	1.3978	0.6532
	Married	751 (62,4)	622 (82.8)	129 (17.2)				
	Divorced	44 (3.7)	35 (79.5)	9 (20.5)				
	Widow	9 (0.7)	8 (88.1)	1 (11.9)				
Religion	Christian	955 (79.4)	829 (86.8)	126 (13.2)	1.4073	1.1624	1.7039	0.0739
	Muslim	222 (18.5)	172 (77.5)	50 (22.5)				
	Traditional	26 (2.0)	22 (87.5)	4 (12.5)				
Employment	Unemployed	435 (36.2)	346 (79.5)	89 (21.5)	1.4318	0.9493	2.1593	0.3823
	Employed	768 (63.8)	677 (88.2)	91 (11.8)				
Income/month (₦)	<20,000	260 (21.6)	213 (81.9)	47 (18.1)	0.8651	0.7495	0.9986	0.3126
	>20,000	943 (78.4)	810 (85.9)	133 (14.1)				

Very Significant ∗∗ TB - Tuberculosis.

The majority of the respondents (65.5 ​%) reported adequate knowledge of TB, in relation to its symptoms and control measures. Individuals having relations or households with TB reported higher TB prevalence than their counterparts without such relationships. Higher TB prevalence was reported among patients with haemoptysis (27.1 ​%) than individuals without blood-stained sputum (15.2 ​%). A significantly higher prevalence of TB was found among individuals living in overcrowded and small-sized rooms/accommodations. HIV-positive individuals recorded higher TB prevalence (16.9 ​%) than HIV-negative patients (14.5 ​%). Smoking was found to be significantly associated with a high TB infection rate. Significantly higher TB prevalence was reported during the dry and dusty season (20.0 ​%) than the wet season (8.2 ​%). High occurrences of TB were reported among patients with noncommunicable diseases in the studied population; diabetes 23.1 ​%, hypertension 14.8 ​%, and liver disease 33.3 ​% (Table 3).

**Table 3:** Risk factors associated with tuberculosis in Ekiti and Ondo States, Nigeria.

Characters		Total	TB Positive (%)	Odd Ratio	Lower Odd Ratio	Upper Odd Ratio	*P*-value
TB Knowledge	No	415	45 (10.8)	0.8832	0.6987	1.1164	0.5961
	Yes	788	135 (17.1)				
Relation with TB	No	1025	131 (12.8)	1.3211	0.9887	1.7654	0.3367
	Yes	178	49 (27.5)				
Household with Cough	No	937	113 (12.1)	0.6014	0.4136	0.8745	0.1743
	Yes	266	67 (25.2)				
Duration of Cough	No Coughing	905	102 (11.3)	1.3323	1.0699	1.6590	0.1908
	≤2 ​Weeks	175	56 (32.0)				
	>2 Weeks	123	22 (17.9)				
Bloody sputum	No	1155	167 (15.2)	1.1490	0.7362	1.7932	0.7550
	Yes	48	13 (27.1)				
Overcrowding (persons/room)	1	120	9 (7.5)	0.7734	0.6890	0.8681	0.0262∗
	2	663	117 (17.6)				
	>2	420	54 (12.9)				
Room size	<10/10 ​ft	417	86 (20.1)	0.5809	0.4874	0.6925	0.0020∗∗
	≥12/12 ​ft	786	94 (12.0)				
HIV Status	Negative	960	139 (14.5)	1.0012	0.7715	1.2993	0.9963
	Positive	243	41 (16.9)				
Smoking	No	1081	159 (14.7)	0.6824	0.5792	0.8040	0.0198∗
	Yes	122	21 (17.2)				
Alcoholism	No	1046	154 (14.7)	0.9616	0.5716	1.6178	0.9401
	Yes	157	26 (16.6)				
Season	Dry & Dusty	691	138 (20.0)	0.4683	0.3584	0.6119	0.0046∗∗
	Rain & Wet	512	42 (8.2)				
NonCommunicable Diseases	None	1114	165 (14.8)	0.8658	0.7612	0.9848	0.2631
	Diabetes	26	6 (23.1)				
	Hypertension	27	4 (14.8)				
	Liver Disease	6	2 (33.3)				
	Others	30	3 (10.0)				

Significant ∗ Very Significant ∗∗ TB - Tuberculosis.

The risk factors identified for tuberculosis prevalence in Ekiti and Ondo States combined were: gender (OR ​= ​0.548, 95 ​% CI ​= ​0.446–0.675, p ​= ​0.004); overcrowding (OR ​= ​0.733, 95 ​% CI ​= ​0.689–0.868, p ​= ​0.026); room size (OR ​= ​0.580, 95 ​% CI ​= ​0.487–0.692, p ​= ​0.002); smoking (OR ​= ​0.682, 95 ​% CI ​= ​0.579–0.804, p ​= ​0.002) and dry and dusty season (OR ​= ​0.468, 95 ​% CI ​= ​0.358–0.612, p ​= ​0.005).

The identified risk factors for MDR in the two Nigerian states were education (OR ​= ​0.739, 95 ​% CI ​= ​0.731–0.745, p ​= ​0.0175), age (OR ​= ​0.846, 95 ​% CI ​= ​0.341–0.850, p ​= ​0.0475), religion (OR ​= ​1.953, 95 ​% CI ​= ​1.953, p ​= ​0.0003), family income (OR ​= ​1.762, 95 ​% CI ​= ​1.751–1.775, p ​= ​0.008), TB knowledge (OR ​= ​1.746, 95 ​% CI ​= ​1.738–1.753, p ​= ​0.0311), household with cough (OR ​= ​3.551; 95 ​% CI ​= ​2.544–3.563, p ​= ​0.0002), duration of cough (OR ​= ​1.636; 95 ​% CI ​= ​1.623–1.648, p ​= ​0.0187), previous TB treatment (OR ​= ​3.640, 95 ​% CI ​= ​3.614–3.666, p ​= ​0.0041), smoking (OR ​= ​1.334, 95 ​% CI ​= ​1.328–1.342, p ​= ​0.0348) and HIV status (OR ​= ​1.85, 95 ​% CI ​= ​p ​= ​0.0065), Table 4.

**Table 4:** Association of MDR-TB with Socio-demographical and Risk factors.

Coefficients	Estimate	Std. Error	z value	Pr (>|z|)	Odds Ratio	UOR	LOR
Education	−0.3030	0.1275	−2.377	0.0175 ∗	0.7386	0.7451	0.7309
Sex	0.1114	0.2050	0.543	0.5869	1.1178	1.1178	0.9106
Age	−0.1678	0.0847	−1.982	0.0475 ∗	0.8455	0.8503	0.8408
Religion	0.6695	0.1851	3.617	0.0003 ∗∗∗	1.9533	1.9638	1.9428
Family Income	0.5669	0.2129	2.664	0.0077 ∗∗	1.7629	1.7749	1.7509
TB knowledge	0.5572	0.2585	2.156	0.0311 ∗	1.7458	1.7532	1.7383
Household with Cough	1.2679	0.3378	3.753	0.0002 ∗∗∗	3.5536	3.5633	2.5439
Duration Coughing	0.4921	0.2093	2.351	0.0187 ∗	1.6357	1.6475	1.6239
Previous treatment	1.2920	0.4497	2.873	0.0041 ∗∗	3.6402	3.6656	3.6148
HIV status	0.5907	0.2172	2.719	0.0065 ∗∗	1.8053	1.8176	1.7430
Smoking	0.2885	0.1367	2.111	0.0348 ∗	1.3344	1.3422	1.3276

Significant ∗ Very Significant ∗∗ Very much Significant∗∗∗.

The antibiotic susceptibility profiles of the 15 Rifampicin resistant and 17 Rifampicin susceptible *M. tuberculosis* isolates tested are presented in Table 5. Rifampicin monoresistant was reported in only one (6.7 ​%) of the rifampicin-resistant isolates, while the remaining 14 (93.3 ​%) were rifampicin polyresistant strains. Two (13.3 ​%) of the MDR-TB isolates were resistant to all the 3 first-line antimycobacterial agents tested (Rifampicin, Isoniazid, and Ethambutol); two (13.3 ​%) were Rifampicin-Isoniazid resistant only, one (6.7 ​%) Rifampicin-Ethambutol resistant combined. The Rifampicin-resistant isolates were all susceptible to the aminoglycosides tested (Amikacin, Capreomycin and Kanamycin); also, high susceptibility of the isolates to the fluoroquinolones (100 ​% for moxifloxacin and 86.7 ​% for Levofloxacin) were recorded. Of the 17 rifampicin-susceptible MTB isolates, 16 (94.1 ​%) were susceptible to all the eight antibiotics tested, while one (5.9 ​%) was susceptible to Rifampicin and Isoniazid but resistant to the rest antibiotics.

**Table 5:** Susceptibility profile of *M. tuberculosis* isolates to antibacterial agents.

Isolates	RIF	INH	ETM	KAN	AMK	CAP	LEV	MOX	PRO/ETO	Susceptibility (%)
MDR-TB										
1	R	R	S	S	S	S	S	S	R	66.7
2	R	R	S	S	S	S	S	S	R	66.7
3	R	R	S	S	S	S	S	S	S	77.8
4	R	R	R	S	S	S	R	S	S	55.6
5	R	R	S	S	S	S	S	S	S	77.8
6	R	R	S	S	S	S	S	S	S	77.8
7	R	S	R	S	S	S	R	S	S	66.7
8	R	S	S	S	S	S	S	S	S	88.9
9	R	R	R	S	S	S	S	S	S	66.7
10	R	R	S	S	S	S	S	S	R	66.7
11	R	R	S	S	S	S	S	S	R	66.7
12	R	R	S	S	S	S	S	S	R	66.7
13	R	R	S	S	S	S	S	S	S	77.8
14	R	R	S	S	S	S	S	S	S	77.8
15	R	R	S	S	S	S	S	S	S	77.8
Susceptibility (%)	0	13.3	80	100	100	100	86.7	100	66.7	71.9
Non MDR-TB										
16	S	S	S	S	S	S	S	S	S	100
17	S	S	S	S	S	S	S	S	S	100
18	S	S	S	S	S	S	S	S	S	100
19	S	S	S	S	S	S	S	S	S	100
20	S	S	S	S	S	S	S	S	S	100
21	S	S	S	S	S	S	S	S	S	100
22	S	S	S	S	S	S	S	S	S	100
23	S	S	S	S	S	S	S	S	S	100
24	S	S	S	S	S	S	S	S	S	100
25	S	S	S	S	S	S	S	S	S	100
26	S	S	S	S	S	S	S	S	S	100
27	S	S	S	S	S	S	S	S	S	100
28	S	S	S	S	S	S	S	S	S	100
29	S	S	S	S	S	S	S	S	S	100
30	S	S	S	S	S	S	S	S	S	100
31	S	S	S	S	S	S	S	S	S	100
32	S	S	R	R	R	R	R	R	R	100
Susceptibility (%)	100	100	100	94.1	94.1	94.1	94.1	94.1	94.1	77.8

S = Susceptible R = Resistance.

Rifampicin (RIF), Isoniazid (INH), Ethambutol (EMB), Kanamycin (KAN), Amikacin (AMK), Capreomycin (CAP), Levofloxacin (LEV), Moxifloxacin (MOX) and Protionamide/Ethionamide (PRO/ETO).

## 4 Discussion

TB is a high-burden disease in Nigeria and many countries of the world, and MDR-TB remains a public health crisis and a health security threat [1].

Age, gender, education, marital status, religion, working station, employment, and income were socio-demographically identified factors that significantly influence TB and MDR-TB infection in Ekiti and Ondo States, Nigeria. A holistic approach focusing on these factors will go a long way in a TB-free society in the understudied environment, in Nigeria, and in Sub-Saharan Africa.

The study showed that young people of 20 years and below recorded lower TB prevalence (11 ​%) than those above 20 years of age (15.7 ​%). Children of the age of 10 years and below recorded 10 ​% TB prevalence, which is lower than the 12 ​% for the age group as contained in the World Health Organization's 2021 ​TB Report [1]. Diagnosing tuberculosis in children is a lot of challenging, because collecting samples for analysis is difficult, especially among children under the age of 8 years [18]. MDR-TB was found in the study to occur more frequently among 31–60-year-olds than both the younger and older age groups; which was in consonance with the report from a previous study that active adults have a higher risk of MDR-TB [19]. MDR-TB at a young age has been attributed to adhere in adhering to TB treatment regime [20].

In this study, the occurrences of TB among male and female participants were 19.3 ​% and 11.0 ​% respectively, with a male-to-female ratio of 1.75:1; which is incongruent with the national and global male-to-female TB ratio of 1.8:1 and 1.75:1 respectively. This gender bias in TB distribution has been attributed physiological and behavioural altitudes of males. There is an abundant immune-related gene on the X-chromosome that tends to have immunity against tuberculosis. The X-chromosome consists of nearly 1100 genes, most of which are immunomodulatory compared to 100 genes on the Y-chromosome. Furthermore, females had stronger immune responses to antigenic reactions, such as vaccination or infection, than males [1].

The socio-cultural activities, risks, roles, behaviours, and professional practices associated with high-risk TB are more associated with males than females, as they can travel more frequently, have more social contacts, and spend more time in social gatherings such as bars, movie theatres, and nightclubs, which may be a permissive environment for TB transmission [18]; just as smoking and alcoholism are more common among males than females [1]. The link between cigarette use and tuberculosis has been well documented [1,21]. The current study found a 17.2% TB incidence among smokers, which is lower than the 22.7 ​% previously reported by Okiki et al. [22] in Ikere-Ekiti, Nigeria, and 26.3 ​% by Kirenga et al. in Kampala, Uganda [23].

There is a wide gap in the seasonal differential of TB prevalence, with 8.2 ​% for the rainy season and 20.0 for the dry season. Seasonal variation has been implicated in the spread of TB in earlier work in Hong Kong by Xu et al. [24] that reported associations of temperature and relative humidity in the incidence of tuberculosis. When infected aerosol of *M. tuberculosis* is discharged by active TB patients, the air-borne particles can easily be inhaled in dry and relative humidity conditions [25].

Viana et al. [25] opined that education should not be underscored on matters relating to tuberculosis, as many authors do, as it has a significant role to play in the control of tuberculosis. This assertion is supported in the present study as a significant association between educational status and the occurrence of MDR (OR ​= ​0.73858; p ​= ​0.0175). Educational attainment often determines socioeconomic status and behavioural patterns related to health issues. Patients with no or little education tend to default or not comply with the treatment regimen [25]. Another major factor for MDR-TB is in-adherence to previous TB treatment with the first-line medication regimen, which often leads to the development of MDR-TB [1]. This study obtained a strong association between MDR and previous TB treatment history.

Religion was identified as a major risk factor for the occurrence of MDR-TB. (OR ​= ​1.41, p ​= ​0.0003) in the study area. A similar study in rural South Africa affirmed that most of the traditional worshipers believed in the spiritual causation of the disease and sought first care from the spiritualist. This religious influence has also been recorded in other countries like Gambia, Tanzania, Kenya, and Malawi [26]. HIV infection is an independent risk factor for MDR-TB (p ​= ​0.006) in the study area. People living with HIV/AIDS have been reported to stand a higher risk of developing active TB with a higher mortality rate than people with negative HIV status [1].

Rapid new diagnostic methods (including Xpert MTB/RIF assay) use rifampicin resistance as a surrogate marker for multidrug-resistant tuberculosis. In the year 2020, 71 ​% of people diagnosed with bacteriologically confirmed pulmonary TB tested positive for rifampicin resistance [1]. In the present study 7.9 ​% (15 of 180) positive TB cases, representing 1.5 ​% (15 of 1203) studied population were rifampicin resistant. Rifampicin monoresistant was reported in only one (6.7 ​%) of the rifampicin-resistant isolates, while the remaining 14 (93.3 ​%) were rifampicin polyresistant strains. Two (13.3 ​%) of the MDR-TB isolates were resistant to all the 3 first-line antimycobacterial agents tested (Rifampicin, Isoniazid, and Ethambutol); two (13.3 ​%) were Rifampicin-Isoniazid resistant only, one (6.7 ​%) Rifampicin-Ethambutol resistant combination. Thapa and co-workers in a study in German-Nepal reported TB resistance to Isoniazid 23 ​%, Rifampicin 17.8 ​%, and Ethambutol 15.6 ​% [27]. Zhu et al. [28] in a multicentre cohort study in China working on isolates from 1613 ​TB patients, reported that 33.2 ​% were resistant to at least one first-line drug: monodrug-resistance (22.1 ​%) - Rifampicin, RIF (0.7 ​%) Isoniazid, INH (12.2 ​%), pyrazinamide, PZA (0.8 ​%), ethambutol, EMB (8.4 ​%); polydrug-resistance (3.0 ​%): INH-EMB (2.4 ​%), PZA-EMB (0.6 ​%); and multidrug: RIF-INH (3.5 ​%), RIF–INH–PZA (1.7 ​%) RIF–INH–EMB (2.2 ​%), RIF–INH–PZA-EMB (0.7 ​%). While Colman and co-workers [29] working on sputum samples from 25 tuberculosis patients at risk for drug resistance in the Republic of Moldova, showed that 17 (74 ​%) MDR (RIF ​+ ​INH resistant), 1 (4 ​%) INH mono-resistant, 2 (9 ​%) RIF mono-resistant and 3 (13 ​%) susceptible to INH and RIF.

The Rifampicin-resistant isolates in the present study were all susceptible to the aminoglycosides tested (Amikacin, Capreomycin and Kanamycin); also, high susceptibility of the isolates to the fluoroquinolones (100 ​% for moxifloxacin and 86.7 ​% for Levofloxacin) were recorded. This finding is in agreement with earlier reports that Kanamycin, Capreomycin, and Moxifloxacin were very effective against *M. tuberculosis* [1]

Patients infected with rifampicin-susceptible MTB strains are always prescribed first-line anti-tuberculosis therapy; hence the strains with resistance to other first-line anti-tuberculosis drugs including isoniazid will be missed and inappropriate treatment given [1,30]. Fash and co-workers [30] in analysing rifampicin-susceptible MTB strains, reported resistance to at least one of the first-line drugs in 27 ​% of isolates. They noted an overall isoniazid resistance of 15.5 ​%, with an isoniazid mono-resistance rate of 4 ​%, and a combined resistance of isoniazid-pyrazinamide-ethambutol to be 1 ​%; while resistance to isoniazid-pyrazinamide-ethambutol-streptomycin was observed in 1.7 ​% of strains. In the present study, all but one of the seventeen rifampicin-susceptible MTB isolates were susceptible to all other antibiotics tested. Only one isolate was resistant to all the antibiotics tested with the exception of Rifampicin and Isoniazid, however, more resistance pattern of Rifampicin-susceptible TB isolates to a variety of antibiotics might have been noticed, as reported by Zhu, if higher numbers of isolates were tested.

In Nigeria, culturing and drug susceptibility testing are rarely carried out on Rifampicin susceptible TB sputum, but only on Rifampicin-resistant TB sputum. Since the treatment of TB is a combination therapy of antimycobacterial agents, this will always result in poor treatment outcomes and continuous transmission of drug-resistant TB. The good news is that the rifampicin-resistant MTB isolates were all susceptible to the aminoglycosides and the fluoroquinolones.

To achieve the WHO goal of eradicating TB by 2030 or reducing it to the barest level, all cases of TB, whether rifampicin-resistant or susceptible, should be subjected to drug susceptibility testing, and the subjects treated according. To achieve this there must be a commitment by the government at all levels and all stakeholders in establishing and funding TB culture centres, and effective monitoring of TB patients.

## Declaration of competing interest

The authors declare no conflict of interest
